# A Latent Profile Analysis of Greek University Students’ Sexting Profiles: Associations with Big Five Personality Traits

**DOI:** 10.1007/s10508-023-02762-9

**Published:** 2023-12-27

**Authors:** Constantinos M. Kokkinos, Christina Krommida, Angelos Markos, Ioanna Voulgaridou

**Affiliations:** Department of Primary Education, School of Education Sciences, Democritus University of Thrace, N. Hili, 68131 Alexandroupolis, Greece

**Keywords:** Sexting, Latent profile analysis, Big Five, Non-consensual, University students, Sexual orientation

## Abstract

The aim of this study was to examine the associations between sexting profiles and five factor model (FFM) personality traits among Greek university students. A sample of 2913 participants predominantly aged between 18 and 25 years (*M* = 22; SD = 3.54; 69.6% females) completed a self-report online questionnaire that measured sexting behaviors and FFM traits. Latent profile analysis identified five distinct profiles which were labeled uninvolved, consensual sexters, non-consensual sexters, unwanted sexters, and highly involved sexters. The different sexting profiles were associated with distinct patterns of FFM trait scores. Specifically, the uninvolved scored higher on agreeableness, conscientiousness, and openness than those highly involved in sexting, consensual, non-consensual, and unwanted sexters. Furthermore, those who sent unwanted sexts were more likely to be emotionally unstable than the consensual sexters and those not involved. These findings indicate that sexting behaviors may be related to certain personality traits and emphasize the importance of considering individual differences when studying sexting behaviors. The practical implications of the findings are discussed.

## Introduction

Sexting is the exchange of messages with suggestive or provocative content via the Internet or smartphones (Morelli et al., 2020). Apart from being considered as a common new normative sexual behavior between adults (i.e., experimental or consensual sexting), it may also encompass behaviors such as forwarding or posting sexual or provocative messages of others without consent (non-consensual dissemination) as well as engaging in sexting under pressure (unwanted sexting; Mori et al., 2020). Sexting is a rather complex and dynamic process in which various individual characteristics influence a person’s decision to participate (Sesar et al., 2019). To date, only a few studies have investigated which personality traits are related to sexting behaviors. Meanwhile, a few studies investigated the role of personality characteristics in sexting behaviors employing the Five Factor Model (FFM; Neuroticism, Extraversion, Openness-to-Experience, Agreeableness, Conscientiousness) as the personality theoretical framework (e.g., Alonso & Romero, 2019; Crimmins & Seigfried-Spellar, 2017; Delevi & Weisskirch, 2013; Olatunde & Balogun, 2017). However, all the available studies investigating the association between sexting and FFM used a variable-centered approach, which explores correlations among variables, disregarding the possibility to distinguish subpopulations among participants with distinct sexting profiles. Although person-centered approaches could obtain profiles of sexters that may differ in their personality traits, no study to our knowledge has used Latent Profile Analysis (LPA) to examine different forms of sexting and personality characteristics. Therefore, the present study sought to address this gap by investigating the nature of distinct sexting profiles and explore FFM traits across the latent classes in a sample of Greek university students.

The high prevalence of sexting among young people has been facilitated by easy access to the Internet and the proliferation of communication technologies in daily life (Walrave et al., 2014). Greece is a country with average Internet and social media penetration compared to the rest of the European countries, whereas in terms of the global average it is well above (79%; approx. 8.3 million users; European average 85%; global average 59%) (We Are Social, 2020). Kokkinos et al. (2014) found in a sample of Greek university students that increased Internet use was positively associated with frequent use of chatrooms, instant messaging, social network sites and cyber-bullying.

### Sexting Profiles

The operationalization of sexting as a multidimensional construct reflecting many sorts of sexting is an important next step. Several researchers have attempted to identify various types of sexting using theoretically developed categories to differentiate between different types of sexters (e.g., senders vs. receivers, or sexts involving pictures vs. sexts involving words). Drouin and Landgraff (2012) discriminated between receiving and sending, as well as visuals and text, in their sample of young adult college students in one of the initial studies. Later, Gordon-Messer et al. (2013) classified their young adult (aged 18–24) participants based on sexting directionality (i.e., non-sexters, two-way sexters, receivers only, and senders only), a classification that Currin et al. (2016) also used to differentiate between sexting behaviors in their sample of adults recruited through Internet advertisements. Morelli et al. (2016), in one of the most recent studies, combined measures of sexting directionality with measures of sexting frequency, content, receiver, medium, and other sexting risk factors to divide their Italian participants (aged 18–30) into three groups: low, moderate, and high sexting users. Overall, these studies have provided a framework for distinguishing between different types of sexters (e.g., senders vs. receivers); however, theoretically-derived categories, while useful to some extent, are artificial and not data-driven, and thus may not always represent naturally-occurring typologies of sexting participants within a given sample. Additionally, yet, little research attention has been given to different profiles of sexting participants regarding their consensus to sexting behavior. Roberts and Ravn (2020) found in a recent qualitative study that participants expressed the delicate, hard, and sometimes opaque process of establishing consent in sexting, as well as the ambiguity inherent in the practice. Furthermore, popular media sexting guidelines frequently advocate for consent in sexting (Hasinoff, 2015), despite the fact that there has been little empirical study examining consensual sexting in particular. While sexting research is still in its early stages, it is crucial to distinguish between sexting behaviors that are coerced, unwanted, or non-consensual and those that occur in consensual relationships.

Most researchers refer to sexting as consensual if it is done voluntarily, without pressure or blackmail, and the sexts are forwarded with the approval of the sexts’ creator (Döring, 2014; Morelli et al., 2016). In the case of consensual sexting, romantic partners seem to exchange sexts more often than friends or strangers (Crimmins & Seigfried-Spellar, 2017; Gámez-Guadix et al., 2015). Furthermore, committed partners sext more often than casual sexual partners (Courtice & Shaughnessy, 2017). Aggravated sexting, on the other hand, entails the presence of malice toward someone who shares sexts or forces someone to share sexts (Wolak & Finkelhor, 2011). This sort of sexting includes two types: non-consensual sexting and unwanted sexting. Non-consensual sexting is the sharing of sexts or provocative photo or video messages to third parties without authorization of the portrayed person (Morelli et al., 2016). Unwanted sexting occurs when a spouse or friend puts pressure on a person to send sexts (Drouin et al., 2015; Van Ouytsel et al., 2017). While consensual sexting seems to have a positive impact among those involved (e.g., dating partners, sexual minorities), research shows that the greatest risk is associated with non-consensual sexting (e.g., Brenick et al., 2017; Lim et al., 2016; Wilkinson et al., 2016). However, the extant research on sexting failed to distinguish between consensual and non-consensual sexting behavior. This study sought to clarify this issue by using LPA to divide individuals into distinct groups that share comparable features in terms of their consensus to sexting.

### Sexting Profiles and Personality

Individual differences in personality are usually studied as risk factors for psychological and behavioral problems. The study of personality traits of those involved in sexting is starting to attract research attention (e.g., Drouin et al., 2018; Morelli et al., 2020). To date, few studies have investigated the role of FFM in relation to both normative and aggravated sexting behaviors (e.g., Morelli et al., 2020). The FFM is a modern and widely accepted taxonomic model used to describe personality traits through five major dimensions: Agreeableness which is associated with increased social behavior; Neuroticism characterized by emotional instability; Conscientiousness referring to ambition and commitment to goals; Extraversion which includes increased sociability and the pursuit of enthusiasm, and finally Openness characterized by creativity and a tendency to seek new and exciting experiences (Panayiotou, et al., 2004).

Extraversion was positively correlated with sexting, while there is ambiguous evidence on the role of Neuroticism, Agreeableness, Conscientiousness, and Openness (e.g., Crimmins & Seigfried-Spellar, 2017; Liu & Zheng, 2020). A high prognostic factor for sending sexual texts is high Extraversion, as it is associated with sociability, optimism, high self-esteem and well-being, the pursuit of enthusiasm, and the most tolerant attitude towards sexuality (Heaven et al., 2000). In the case of sexual photos and videos, high Neuroticism and low Agreeableness traits seem to be implicated (Delevi & Weisskirch, 2013).

Comparing adults involved in sexting and those who abstain, Crimmins and Seigfried-Spellar (2017) found that the personality traits of sexters include high Extraversion and low Conscientiousness and Agreeableness. The last two traits mainly describe people with impulsivity, irresponsibility, lack of long-term goals, and antisocial behavior (Jones et al., 2011). However, Drouin et al. (2018) found that individuals scoring high on Conscientiousness were more likely to expect sexual activity after sexting with offline romantic partners. A recent study with Chinese adults (Liu & Zheng, 2020) found that those high in Extraversion, Openness, Sexual Narcissism (i.e., sexual exploitation, low sexual empathy, and sexual exhibitionism), Narcissism (i.e., sense of superiority), and low in Neuroticism, were more likely to engage in Internet Sexual Activity of any form. To acquire a more comprehensive view of the association between personality and sexting among university students studies from similar research with adolescents were also taken into consideration. Notably, the only traits reported consistently being associated with sexting are high Extraversion and low Conscientiousness (Alonso & Romero, 2019; de Santisteban & Gámez-Guadix, 2018; Gámez-Guadix et al., 2017). However, only two studies reported low Agreeableness (Alonso & Romero, 2019; Gámez-Guadix et al., 2017), and one reported high Neuroticism being associated with sexting as well (Gámez-Guadix et al., 2017).

Nevertheless, two studies have investigated the link between personality traits and sexting in mixed aged groups. A study conducted in Nigeria among students aged 10–24 by Olatunde and Balogun (2017) found that individuals with high levels of Extraversion were more likely to participate in sexting, problematic cell phone use, and engage in risky sexual behaviors, such as having multiple sexual partners. The authors identified these traits as strong predictors for engaging in sexting. A more recent cross-cultural study in 20 European countries explored the relationship between personality traits and high-risk and aggravated sexting behaviors, including sexting during substance use, sexting with strangers met online, sharing sexts of others without their consent, and engaging in unwanted sexting among individuals aged 13–30 years (Morelli et al., 2020). The study revealed that individuals with low levels of Neuroticism and Conscientiousness were more likely to participate in all three sexting behaviors. Furthermore, high-risk sexting behaviors were positively correlated with Extraversion, while negatively correlated with Agreeableness. Finally, non-consensual sexting was negatively correlated with Openness. Moreover, Clancy et al. (2019) found significant links between the dark triad personality traits (i.e., Machiavellianism, Narcissism, and Psychopathy) and non-consensual sharing of sexts. These traits are frequently associated with manipulation, egoism, aggressiveness, and emotional coldness (Koehn et al., 2019). In a meta-analytic review of the relationship between the dark triad and FFM personality taxonomy, Schreiber and Marcus (2020) found that all three dark traits were negatively correlated with Agreeableness, while Psychopathy and Machiavellianism were also negatively associated with Conscientiousness. Conversely, Narcissism demonstrated high positive correlations with Extraversion, and to a lesser degree, with Openness, which were not as evident for Psychopathy and Machiavellianism. Therefore, non-consensual sexting is likely to be linked with low levels of Agreeableness and Conscientiousness, as well as high levels of Extraversion and Openness.

### The Role of Gender and Sexual Orientation

When gender is considered, the results have indicated that there is a significant difference in sending sexual texts between genders. Specifically, men who are in a romantic relationship and score high in Extraversion and Neuroticism (Delevi & Weisskirch, 2013) are more likely to sext, suggesting that being social and talkative, and at the same time sensitive, shy, and overly dependent on others may contribute to sexting behavior (Costa & McCrae, 2008). Furthermore, Drouin et al. (2018) reported that single men scoring high on Extraversion and low on Conscientiousness were more likely to anticipate that their online dating partners would fulfill their sexual fantasies earlier expressed in sexual messages. There is no research relating personality traits and sexting with sexual orientation. However, heterosexuals and people of different sexual orientation seem to have dissimilar personality characteristics (Bem, 1996), but these differences are reduced as age increases (Allen & Robson, 2020). Recent studies concluded that non-heterosexuals reported higher levels of Openness than heterosexuals (Allen & Robson, 2020; Allen & Walter, 2018).

### The Present Study

Given that sexting is generally considered a behavior of the modern digital age, especially among youth, the present study aimed to investigate the relationship between various forms of sexting and the FFM among Greek university students. Prior research on sexting has mainly adopted a variable-centered approach to investigate the associations of FFM with each form of sexting, while neglecting the potential differences in the personality traits of individuals classified in distinct sexting profiles. To address this limitation, the present study adopted a person-centered approach through LPA to categorize participants into distinct groups based on their patterns of sexting behavior. This approach enables an in-depth exploration of how students in different sexting profiles compare to each other with regard to their personality traits. Based on the available literature at least two groups will emerge from the LPA analysis, representing consensual and aggravated sexting behaviors. Further, it is expected that the aggravated sexting profile may be further subdivided into non-consensual and unwanted sexting groups.

Moreover, a second goal of the present study was to investigate whether the FFM personality traits could differentiate students’ membership in each sexting profile. Thus, while different forms of sexting were used to form the latent profiles, whether these classes of individuals are associated with distinct personality traits (e.g., participants with higher extraversion scores presented a higher likelihood of membership into the highly involved profile relative to all other profiles) was also examined. This study represents the first research in Greece that explores this relationship, encompassing not only consensual but also non-consensual and unwanted sexting among participants with different sexual orientation and gender identities. The study findings are expected to provide insight into the development of sex education programs aimed at enlightening young people on the risks associated with online sexual behaviors. Additionally, they are expected to inform the development of prevention programs that will provide the requisite skills for resisting sexual coercion, particularly in the context of unwanted sexting.

## Method

### Participants and Procedure

Participants were 2913 Greek public university students. A substantial majority, constituting 90% of our sample, were aged between 18 and 25 years. Furthermore, 99% of the participants were under the age of 31. It’s noteworthy that only fifteen participants, representing a mere 0.5% of the sample, were aged above 31. The mean age was 22 years (SD = 3.54) with 69.6% being females. The majority of participants were exclusively heterosexual (76.8%; *n* = 2238), 97.6% of them were Greeks, who reported having engaged in sexting during the last year.

This study was approved by the Institution’s Ethics Committee. Participants were recruited via an anonymous online questionnaire through Facebook posts to online Greek student communities, during May and June 2020 for a period of one month and gave their informed consent before participating. The recruitment process used snowball sampling and the participation was voluntary and confidential, without personal benefit.

### Measures

#### Demographics

Participants reported their age, gender, nationality, and level of education.

#### Sexual Orientation

Participants reported their sexual orientation via a modified Kinsey Scale (Kinsey et al., 1948), ranging from 1 = *exclusively heterosexual*) to 5 = *exclusively homosexual*. Then, they were categorized into two groups: exclusively heterosexual (who answered 1) and non-exclusive heterosexual (who answered 2–5).

#### Sexting Behaviors

To measure the three forms of sexting (i.e., consensual, non-consensual, and unwanted), 12 items from the Greek Sexting Behaviors Scale (G-SBS) developed by Kokkinos and Krommida (2022) were used for consensual sexting, along with 11 items from the Sexting Behaviors Scale developed by Morelli et al. (2016) for non-consensual and unwanted sexting. Participants were asked to rate the frequency of their involvement in each sexting behavior on a 5-point Likert scale, ranging from 1 = *never* to 5 = *frequently or daily*. Specifically, three items were used to assess the frequency of engaging with text messages and nine to assess the frequency of engaging with photo/video messages. The total sexting score was calculated by averaging the 12 items of the G-SBS and had a Cronbach’s alpha of 0.85. Eight items assessed the non-consensual dissemination of sexts, consisting of forwarding (4 items) and posting sexts (4 items) of someone else (i.e., a romantic partner, ex-partner, friend, and a person met on the internet) without his/her consent (e.g., “How often do you post-sexually or provocative photos or videos where the person pictured is one of the following, without his or her consent on social media?”). The total non-consensual sexting score was calculated by averaging the 8 items and had a Cronbach’s alpha of 0.76. Three items assessed sexting under pressure by a romantic partner, friends, or a stranger (e.g., “How often do you receive, send, or post-sexual or provocative text messages, photos/videos via mobile devices, the internet or social media because he pressured you?”; Cronbach’s alpha was 0.60).

#### Personality

The Greek translation of the NEO Five Factor Inventory (NEO-FFI; Panayiotou et al., 2004) was used to measure personality. A 5-point Likert scale ranging from 1 = *Strongly disagree* to 5 = *Strongly agree*, was used to rate each of the 60 items. Each personality factor showed satisfactory reliability, with Cronbach’s alpha values ranging from.69 to.86 (i.e., Neuroticism 0.82, Extraversion 0.76, Agreeableness 0.71, Conscientiousness 0.86, and Openness 0.69—with the exclusion of an item).

### Data Analysis Plan

Bivariate correlations were calculated among variables using Pearson’s *r* coefficient. Latent profile analysis in Mplus 8.6 (Muthén & Muthén, 2017) was used to identify participant groups based on their scores on consensual, non-consensual and unwanted sexting. LPA seeks to identify subpopulations of participants, referred to as profiles, characterized by distinct configurations on a series of indicators (in the current study, the three forms of sexting) (Morin & Litalien, 2019). The model was estimated for the range of 1–8 latent classes. A total of 5000 random sets of starting values, 1000 iterations and the 200 best solutions were retained for final optimization (Hipp & Bauer, 2006). The optimal number of profiles was determined by considering both the statistical adequacy of the alternative solutions, as well as their interpretability (Marsh et al., 2009). The following fit indices were used to help guide this decision: the Akaike Information Criterion (AIC), the Bayesian Information Criterion (BIC), the sample-size-adjusted BIC (SSA-BIC), the Lo–Mendell–Rubin likelihood ratio test (LMR), the bootstrap likelihood ratio test (BLRT), and entropy. Lower AIC, CAIC, BIC, and ABIC values indicate better-fitting models. A non-significant LMR or BLRT *p-*value suggests that a model with one fewer class is preferred (Lo et al., 2001). An entropy value larger than 0.80 indicates good classification quality. Once the optimal model was selected, chi-square tests were conducted to investigate associations between gender, sexual orientation and latent profile membership, and analyses of variance (ANOVAs) followed by Scheffé’s post-hoc test were conducted in IBM SPSS 23 to examine the differences in the mean FFM scores as a function of latent class membership.

## Results

### Descriptive Statistics and Group Differences

Overall, participants reported more frequent consensual sexting, whereas their mean scores in unwanted and consensual sexting were low. Specifically, 68% of the participants had sexted at least once in the last 12 months, 19% had disseminated non-consensual sexts, and 17% had engaged in unwanted sexting. A large number of females reported having sexted at least once in the past year (70%) and engaged in unwanted sexting (20%) in comparison with males (63%; 10%). However, more males reported engaging in non-consensual sexting (22%) than females (17%). Regarding sexual orientation, the prevalence of non-heterosexuals was higher for engaging in all three sexting behaviors than that of heterosexuals.

Gender differences in the three forms of sexting were estimated using independent samples t-tests. Males reported significantly more frequent involvement than females in consensual *t*(874.54) = 7.56, *p* < 0.001, (*M*_M_ = 2.18, SD_M_ = 0.67 and *M*_F_ = 1.93, SD_F_ = 0.55, respectively), as well as in non-consensual sexting *t*(813.39) = 3.02, *p* = 0.003, (*M*_M_ = 1.12, SD_M_ = 0.29 and *M*_F_ = 1.08, SD_F_ = 0.22, respectively). No statistically significant gender differences were found in unwanted sexting.

With regards to sexual orientation differences, non-heterosexuals compared to heterosexuals reported more frequent exchange of sexts *t*(789.84) = − 6.64, *p* < 0.001, (M_NH_ = 2.16, SD_NH_ = 0.66 and M_H_ = 1.94, SD_H_ = 0.56, respectively), as well as more frequent involvement in unwanted sexting *t*(695.70) =  − 3.07, *p* = 0.002, (M_NH_ = 1.18, SD_NH_ = 0.41 and M_H_ = 1.12, SD_H_ = 0.29, respectively). No statistically significant differences were evidenced for non-consensual sexting.

Intercorrelations between consensual, non-consensual and unwanted sexting were positive but low to moderate (*r* = 0.25–0.33, *p* < 0.01; Table 1). Low correlations were also observed between all Big-5 personality traits and consensual (*r* = 0.00 to − 0.02, *p* < 0.01), non-consensual (*r* = 0.03 to − 0.08, *p* < 0.01), and unwanted sexting (*r* = 0.01–0.10, *p* < 0.01), with somewhat stronger correlations for agreeableness (*r* = − 0.08 to − 0.15, *p* < 0.01).

**Table 1:** Means, standard deviations, and intercorrelations of sexting forms and Big-5 factors

	*M*	SD	(1)	(2)	(3)	(4)	(5)	(6)	(7)
CS (1)	2.00	.60							
NCS (2)	1.09	.24	.25						
US (3)	1.14	.33	.25	.33					
O (4)	3.57	.58	− .01	− .08	− .04				
C (5)	3.57	.67	.00	− .08	− .05	.04			
E (6)	3.13	.57	.08	.03	.01	.05	.34		
A (7)	3.47	.53	− .12	− .15	− .08	.17	.09	.22	
N (8)	2.94	.67	− .02	.03	.10	.05	− .33	− .46	− .13

*CS* consensual sexting, *NCS* non-consensual sexting, *US* unwanted sexting, *O* openness, *C* conscientiousness, *E* extraversion, *A* agreeableness, *N* neuroticism, *N* = 1967; Correlations |.08| are significant at *p* < .01; Likert scale range for each variable = 1–5

### Latent Profile Analysis

**Fig. 1:**
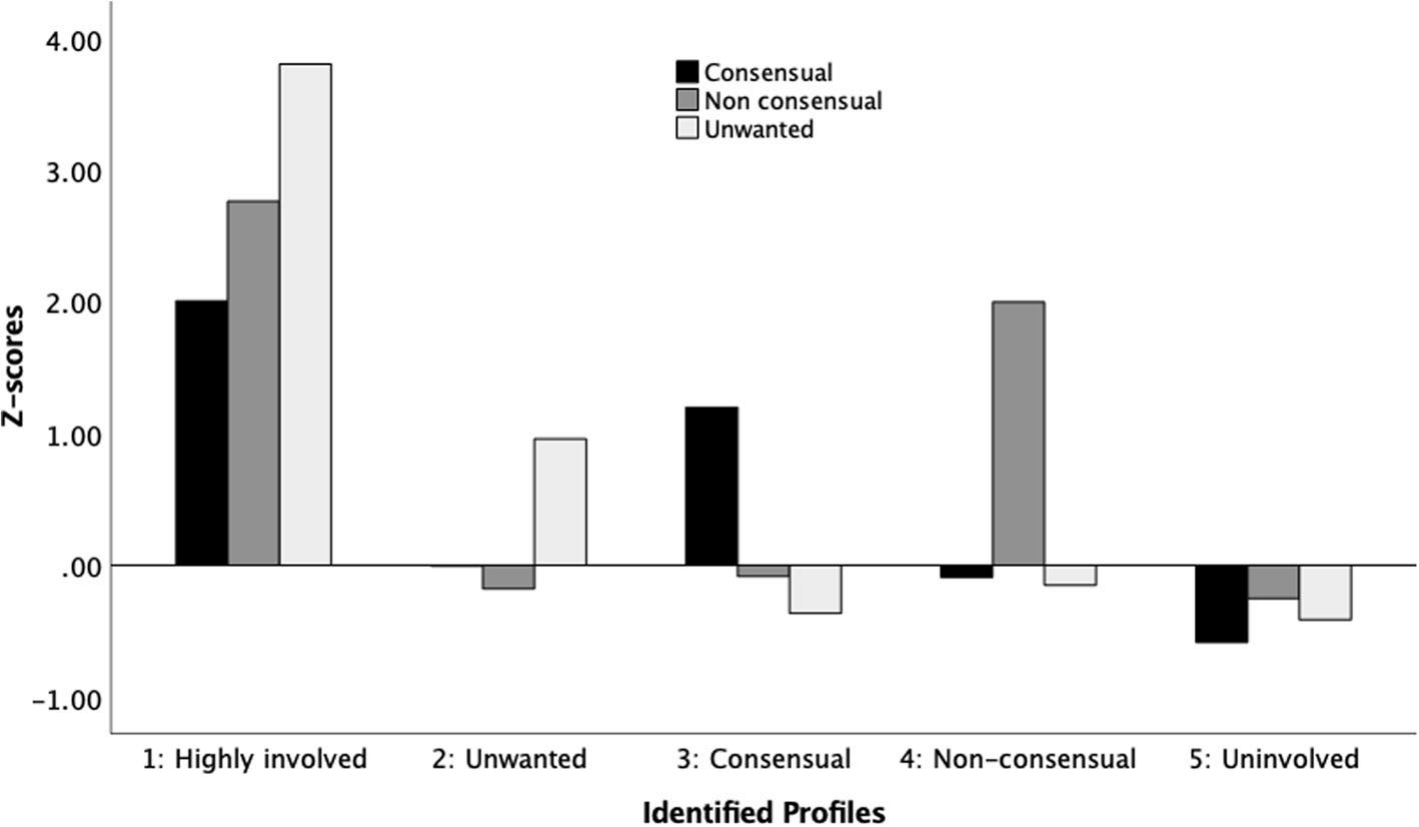
Latent profiles of sexting

To identify the optimal number of groups to retain, models with one to eight classes were estimated using LPA. The AIC and BIC statistics increased from class 5 (AIC = 1855.95, BIC = 1978.8) to class 6 (AIC = 3292.33, BIC = 3437.52) and decreased from class 4 (AIC = 3194.2, BIC = 3294.72) to class 5. In addition, the LMR and BLRT statistics fell out of significance for the six-class model (*p* = 0.64 and *p* = 0.85, respectively). Thus, the five-class model better represented the data. The mean posterior probability scores ranged from 0.90 to 0.99 and the entropy value was 0.84, suggesting that the identified classes were well separated. Figure 1 shows standardized *z*-scores by profile on each grouping variable. As presented in Fig. 1, the identified profiles reflect both quantitative differences (i.e., level differentiation) and qualitative differences (i.e., shape-differentiation). Profile 1 participants (*N* = 50, 2.5%) were characterized by the highest scores in all three sexting forms and were labelled as highly involved. Individuals classified in this profile report that they disseminate, consisting of forwarding or posting sexts (i.e., text, photo or video messages) of someone else with or without his/her consent. In Profile 2 (*N* = 403, 20.5%), participants presented higher scores in unwanted sexting, namely engaging in sexting under pressure by a romantic partner, friends, or a stranger, Profile 3 participants (*N* = 410, 20.8%) had higher scores in consensual sexting, done voluntarily, without pressure or blackmail, and the sexts are forwarded with the approval of the sexts’ creator, and Profile 4 participants (*N* = 109, 5.5%) had higher scores in non-consensual sexting including the sharing of sexts or provocative photo or video messages to third parties without authorization of the portrayed person. Participants in the fifth, dominant profile (*N* = 995, 50.6%) were classified as uninvolved.

### Group Distribution among Students of Different Gender and Sexual Orientation

Chi-square tests showed that subjects in the five profiles differed in terms of gender (Table 2), with males being overrepresented in the highly involved and the consensual profiles, whereas females were more prevalent in the unwanted sexters profile.

**Table 2:** Crosstabulation between gender and Latent Profile Analysis profiles (%)^a^

	LPA profiles					Total *N* (%)
	Highly involved *n* (%)	Unwanted sexters *n* (%)	Consensual sexters *n* (%)	Non-consensual sexters *n* (%)	Uninvolved *n* (%)	
Male	25 (4.5)	57 (10.2)	172 (30.7)	33 (5.9)	273 (48.7)	560 (100)
Female	25 (1.8)	346 (24.6)	238 (16.9)	76 (5.4)	722 (51.3)	1407 (100)
Total	50 (2.5)	403 (20.5)	410 (20.8)	109 (5.5)	995 (50.6)	1967 (100)

*χ*^2^(4, *Ν* = 1967) = 89.28, *p* < .001, Cramer’s *V* = .213)

Furthermore, significant differences in profile membership were observed according to sexual orientation (Table 3), with non-heterosexuals being more prevalent in the highly involved profile and the consensual profile, whereas heterosexuals were overrepresented in the uninvolved profile.

**Table 3:** Crosstabulation between sexual orientation and Latent Profile Analysis profiles

	LPA Profiles					Total
	Highly involved *n* (%)	Unwanted sexters *n* (%)	Consensual sexters *n* (%)	Non-consensual sexters *n* (%)	Uninvolved *n* (%)	*n* (%)
Heterosexual	27 (1.9)	290 (19.9)	271 (18.6)	80 (5.5)	786 (54.1)	1454 (100)
Non-Heterosexual	23 (4.5)	113 (22.0)	139 (27.1)	29 (5.7)	209 (40.7)	513 (100)
Total	50 (2.5)	403 (20.5)	410 (20.8)	109 (5.5)	995 (50.6)	1967 (100)

*χ*^2^(4, *Ν* = 1967) = 37.42, *p* < .001, Cramer’s *V* = .138

### Profile Differences in Terms of Personality Characteristics

One-way ANOVAs followed by Scheffe’s post-hoc test were used to examine the differences in the mean personality subscale scores as a function of latent class membership, with the most likely class membership as the between-groups variable. Results showed that mean personality scores significantly differed in terms of profile membership, in Agreeableness (*F*(4, 1966) = 10.5, *p* < 0.001, *η*^2^ = 0.021), Neuroticism (*F*(4, 1966) = 8.4, *p* < 0.001, *η*^2^ = 0.017), Openness (*F*(4, 1966) = 4.7, *p* < 0.001, *η*^2^ = 0.010), and Conscientiousness (*F*(4, 1966) = 4.5, *p* = 0.001, *η*^2^ = 0.009) (Table 4).

**Table 4:** Participant profile differences in personality

	Profiles											
	Highly involved		Unwanted sexters		Consensual Sexters		Non-consensual sexters		Uninvolved		ANOVA (df = 4, 1966)	
	(*n* = 50)		(*n* = 403)		(*n* = 410)		(*n* = 109)		(*n* = 995)			
	*M*	SD	*M*	SD	*M*	SD	*M*	SD	*M*	SD	*F*	*p*
N	3.05	.66	3.10	.66	2.87	.67	2.98	.65	2.90	.68	8.46	.000
E	3.28	.61	3.09	.55	3.16	.56	3.11	.55	3.13	.59	1.56	.182
A	3.16	.58	3.50	.47	3.40	.54	3.32	.56	3.51	.53	10.58	.000
O	3.42	.67	3.52	.65	3.57	.65	3.38	.61	3.61	.68	4.51	.001
C	3.45	.61	3.57	.54	3.54	.59	3.39	.63	3.61	.57	4.78	.001

*N* neuroticism, *E* extraversion, *A* agreeableness, *O* openness, *C* conscientiousness; Likert scale range for the variables = 1–5

The results indicated that unwanted sexters (*M* = 3.10, SD = 0.66) scored higher on Neuroticism than consensual sexters (M = 2.87, SD = 0.67) and those uninvolved (*M* = 2.90, SD = 0.68). Furthermore, the uninvolved (*M* = 3.61, SD = 0.57) scored higher on Conscientiousness than the non-consensual sexters (*M* = 3.39, SD = 0.63) and higher on Openness (*M* = 3.61, SD = 0.68) than the consensual sexters (*M* = 3.57, SD = 0.65). Regarding Agreeableness, the uninvolved (*M* = 3.51, SD = 0.53) scored higher than the non-consensual (*M* = 3.32, SD = 0.56), the consensual (*M* = 3.40, SD = 0.54) and the highly involved sexters (*M* = 3.16, SD = 0.58). In the same trait, the highly involved scored lower than the unwanted sexters (*M* = 3.50, SD = 0.47).

## Discussion

The purpose of the present study was to examine the association between diverse forms of sexting and the FFM-based personality traits in a large sample of public Greek university students. In addition, this is the first study that relied on LPA to identify subgroups of sexters characterized by distinct personality traits. Results showed that two-thirds of the participants had engaged in consensual sexting, while only one-fifth had participated in non-consensual and unwanted sexing during the past year. Nonetheless, the findings also corroborate that non-consensual and unwanted sexting remains a relatively common incident for young adults (Clancy et al., 2019). Furthermore, they suggest that modern digital culture perceives consensual sexting as a way of expressing one's sexuality (Mori et al., 2020).

Consistent with previous research (e.g., Klettke et al., 2019; Ross et al., 2019), males reported a more frequent involvement in both consensual and non-consensual sexting than females. This observation can be explained by the possibility that men utilize sexting as a means of finding a sexual partner (Delevi & Weisskirch, 2013). Furthermore, men are more inclined to visit sex sites frequently and converse with strangers than women (Liu & Zheng, 2020). Additionally, men are more prone to send non-consensual messages of a sexual or provocative nature to third parties, ostensibly to cause harm to an ex-partner (Walker & Sleath, 2017; Wilkinson et al., 2016) or to control an ex-partner by using psychological violence (Cole et al., 2020). These results provide a rationale for the increased incidence of vindictive sexting among females (Branch et al., 2017), with their sexual images often appearing on non-consensual pornography websites, along with nearly one in five women’s names (Uhl et al., 2018). Non-heterosexuals reported more frequent involvement in consensual and unwanted sexing than heterosexuals consistent with prior research (e.g., Galovan et al., 2018; Olatunde & Balogun, 2017). This possibly suggests that people of different sexual orientation use the Internet and the relative anonymity it provides, to search for sexual partners and avoid public stigmatization (Galovan et al., 2018). In a recent study conducted by Holmes et al. (2021) non-heterosexuals reported that sexting had more positive effects on them than heterosexuals did. However, non-heterosexuals have increased exposure to Internet-related risks, such as victimization or the pressure to engage in unwanted sexting (Gámez-Guadix et al., 2015). Further investigation on the effects of sexting on non-heterosexuals is required.

LPA results showed the existence of five distinct profiles of university students based on their scores on the three forms of sexting. The first group was characterized by the highest scores in all three sexting forms and were labelled as highly involved. Participants in the second group had higher scores in unwanted sexting, participants in the third group in consensual sexting, and participants of the fourth group had higher scores in non-consensual sexting. A final group, the uninvolved (the most prevalent profile), showed lower scores on all sexting forms. The results demonstrated that the uninvolved (self-disciplined, responsible and reliable as well as open to experience and agreeable) were more likely to score higher on Agreeableness, Conscientiousness, and Openness compared with those highly involved in sexting, consensual, non-consensual, and unwanted sexters (i.e., distant, unfriendly, and uncooperative). In all, this group is characterized by positive personality traits such as self-discipline, responsibility, reliability, openness to new experiences, and agreeableness. These individuals tend to be organized, friendly, cooperative, and open to trying new things. Moreover, those who send unwanted sexts were more likely to be emotional unstable (i.e., high Neuroticism) compared to the consensual sexters and those not involved. In contrast, individuals highly involved in sexting, regardless of the nature of their sexting behaviors (consensual, non-consensual, or unwanted), were found to exhibit personality traits associated with being distant, unfriendly, and uncooperative. They scored lower on Agreeableness, Conscientiousness, and Openness, suggesting that they might be less considerate, organized, cooperative, and open to new experiences compared to the uninvolved group.

Specifically, highly involved and unwanted sexters are more likely to report low Conscientiousness, indicating that they are more likely to face problems in their interpersonal relationships. Non-consensual sexters scored low on Openness, Conscientiousness, and Agreeableness, which is partly consistent with previous results (Morelli et al., 2020). Low scores on both Agreeableness and Conscientiousness are associated with low empathy and rudeness, unreliability, along with antisocial and abusive behaviors (Karl et al., 2010). Additionally, low Openness is an indicator of limited ability to recognize the feelings of others (Costa & McCare, 1992). The findings of the present study show that individuals who disseminate non-consensual sexual or provocative messages with these personality traits are more likely to act impulsively and irresponsibly with a tendency for aggressive behaviors in retaliation for their self-interests.

The findings demonstrate that those classified in the unwanted and highly involved sexting profile scored high on Neuroticism and low on Agreeableness in line with prior research (Morelli et al., 2020). Indeed, high Neuroticism and low Agreeableness have been associated with high-risk behaviors (i.e., smoking, substance use), depression, but also acting recklessly when upset (Settles et al., 2012). Individuals high in Neuroticism tend to be emotionally unstable and are likely to seek online social contact to fight loneliness (Hughes et al., 2012). Unwanted sexting is a dangerous and impulsive behavior. The present findings showed that young adults with high Neuroticism, and low Agreeableness are more likely to exchange sexual or provocative messages for fun, without considering the possibility of peril, succumb to pressures, since these individuals are usually insecure, reckless and helpless with a lack of self-discipline and a lack of externalization of their negative emotions.

Surprisingly, Extraversion was not found to differentiate participants’ membership in the five distinct sexting profiles. As high Extraversion indicates the tendency towards high sociability and reveals the search for novelty and excitement through the exchange of sexual or provocative messages (e.g., Delevi & Weisskirch, 2013) it may be a predicting factor of youth’s engagement in sexting. These findings are partly in line with international evidence (e.g., Alonso & Romero, 2019; Crimmins & Seigfried-Spellar, 2017), indicating that sexting serves the needs of todays’ youth, as a way of communicating and socializing with others.

Lastly, it was investigated whether gender and sexual orientation were associated with profile membership. Regarding gender, males had a higher likelihood of belonging to the highly involved and the consensual sexting profiles. This is consistent with previous research that indicated that men are more likely to engage in sexting behaviors as they are less likely to experience negative consequences (Cornelius et al., 2020). A surprising finding of this study was that sexual orientation was related to an involvement in sexting, with non-heterosexuals scoring high on sexting. A possible explanation for this is that the Internet is a medium through which the lesbian, gay, bisexual, and/ or transgendered community can interact and maintain intimate relationships with others without fear of negative social consequences, and thus this group may participate more in sexting behaviors as a way of expressing a sexual orientation which is still socially repressed (Gámez-Guadix et al., 2017). Future studies could further explore the association between sexual orientation and sexting.

This study is subject to limitations that should be acknowledged. First, the use of self-report questionnaires may result in socially desirable responses, suggesting that future studies should consider utilizing both quantitative and qualitative methods to comprehensively investigate sexting. Another limitation was the use of a nonrandom sample of university students, which limits the external validity of the findings. Future research should employ a more representative sample of the general population to enhance the generalizability of the findings on the association between personality and sexting. Nevertheless, as sexting is prevalent among university students (i.e., emerging adults), more attention should be paid to this population. Further, due to its cross-sectional nature, understanding the causal relationships among the variables can be challenging for future longitudinal research. Lastly, this study found small to medium effect size differences in personality traits in all forms of sexting, which suggests that the results should be interpreted with caution.

Despite the above limitations, these findings are an interesting contribution to the sexting literature, as no such comparison has been made to date suggesting that a specific personality profile of sexters exists in comparison with the general population. Therefore, strengths of the study should also be noted. It is the first study to investigate all three forms of sexting in Greece and particularly in a large sample of Greek university students from public universities. Similarly, it is the first study to investigate personality characteristics associated with all three forms of sexing, but also the first to investigate LPA profiles associated with personality characteristics. In addition, while a self-report online questionnaire has some weaknesses, the anonymity of the survey provided minimized the concerns about confidentiality.

From the findings of this study, some important practical applications seem to emerge. At first, there is a need for sex education programs for young people on dangerous online sexual behavior and especially with non-consensual dissemination of sexts and unwanted sexting. Thus, teaching necessary skills to deal with online sexual harassment is of the utmost importance. In addition, awareness raising programs on the negative effects of sexting and the risks involved should be developed. Seminars for students on stress management in their interpersonal relationships and exposing their body to the Internet in order to satisfy their partners, should also be conducted. These programs would be more effective if applied to adolescents as well, as at this age, personality is influenced by culture and social environment along with a constant search for identity. Therefore, intervention programs implemented at such ages can bring positive results, encouraging the creation of critical thinking and developing the ethics of reasoning. Unfortunately, most Greek schools recognize the problem only when a student engages in dangerous and illegal behaviors through sexting. Teachers should therefore be directly informed about this phenomenon and of the negative consequences it brings to teenage students.

### Conclusions

In all, the study found that most participating university students engaged in consensual sexting as a way of expressing their sexuality and communicating with others. However, some also experienced non-consensual and unwanted sexting, which had negative consequences for their well-being and relationships. The study also identified five profiles of sexters based on their involvement in different forms of sexting and their personality traits. It was also found that gender and sexual orientation influenced sexting behaviors and outcomes, with males and non-heterosexuals being more involved in sexting than females and heterosexuals.

## Data Availability

Due to ethical concerns about confidentiality, the data used in the research are not available. The materials used in the research are available upon reasonable request.

## References

[CR1] Allen MS, Robson DA (2020). Personality and body dissatisfaction: An updated systematic review with meta-analysis. Body Image.

[CR2] Allen MS, Walter EE (2018). Linking big five personality traits to sexuality and sexual health: A meta-analytic review. Psychological Bulletin.

[CR3] Alonso C, Romero E (2019). Sexting behaviours in adolescents: Personality predictors and psychosocial outcomes in a one-year follow-up. Annals de Psicología/annals of Psychology.

[CR4] Baumgartner SE, Sumter SR, Peter J, Valkenburg PM, Livingstone S (2014). Does country context matter? Investigating the predictors of teen sexting across Europe. Computers in Human Behavior.

[CR5] Bem DJ (1996). Exotic becomes erotic: A developmental theory of sexual orientation. Psychological Review.

[CR6] Branch KA, Hilinski-Rosick CM, Johnson E, Solano GB (2017). Revenge porn victimization of college students in the United States: An exploratory analysis. International Journal of Cyber Criminology.

[CR7] Brenick A, Flannery KM, Rankin E, Wright M (2017). Victimization or entertainment? How attachment and rejection sensitivity relate to sexting experiences, evaluations, and victimization. Identity, sexuality, and relationships among emerging adults in the digital age.

[CR8] Clancy ME, Klettke B, Hallford JD (2019). The dark side of sexting—factors predicting the dissemination of sexts. Computers in Human Behavior.

[CR9] Cole T, Policastro Ch, Crittenden C, McGuffee K (2020). Freedom to post or invasion of privacy? Analysis of U.S. revenge porn state statutes. Victims & Offenders.

[CR10] Cornelius TL, Bell KM, Kistler T, Drouin M (2020). Consensual sexting among college students: The interplay of coercion and intimate partner aggression in perceived consequences of sexting. International Journal of Environmental Research and Public Health.

[CR11] Costa, P., & McCrae, R. (2008). The revised NEO Personality Inventory (NEO-PI-R). In G. J. Boyle, G. Matthews, & D. H. Saklofske (Eds.), *The SAGE handbook of personality theory and assessment* (Vol. 2: *Personality measurement*) (pp. 179–198). Sage Publications. 10.4135/9781849200479.n9

[CR12] Costa PT, McCrae RR (1992). The five-factor model of personality and its relevance to personality disorders. Journal of Personality Disorders.

[CR13] Courtice EL, Shaughnessy K (2017). Technology-mediated sexual interaction and relationships: A systematic review of the literature. Sexual and Relationship Therapy.

[CR14] Crimmins DM, Seigfried-Spellar KC (2017). Adults who sext: Exploring differences in self-esteem, moral foundations, and personality. International Journal of Cyber Criminology.

[CR15] Currin JM, Jayne CN, Hammer TR, Brim T, Hubach RD (2016). Explicitly pressing send: Impact of sexting on relationship satisfaction. American Journal of Family Therapy.

[CR16] de Santisteban P, Gámez-Guadix M (2018). Prevalence and risk factors among minors for online sexual solicitations and interactions with adults. Journal of Sex Research.

[CR17] Delevi R, Weisskirch SR (2013). Personality factors as predictors of sexting. Computers in Human Behavior.

[CR18] Döring N (2014). Consensual sexting among adolescents: Risk prevention through abstinence education or safer sexting. Cyberpsychology: Journal of Psychosocial Research on Cyberspace.

[CR19] Drouin M, Landgraff C (2012). Texting, sexting, and attachment in college students’ romantic relationships. Computers in Human Behavior.

[CR20] Drouin M, Ross J, Tobin E (2015). Sexting: A new, digital vehicle for intimate partner aggression?. Computers in Human Behavior.

[CR21] Drouin M, Hernandez E, Wehle SMJ (2018). “Tell me lies, tell me sweet little lies:” Sexting deception among adults. Sexuality & Culture.

[CR22] Galovan AM, Drouin M, McDaniel BT (2018). Sexting profiles in the United States and Canada: Implications for individual and relationship well-being. Computers in Human Behavior.

[CR23] Gámez-Guadix M, Almendros C, Borrajo E, Calvete E (2015). Prevalence and association of sexting and online sexual victimization among Spanish adults. Sexuality Research and Social Policy.

[CR24] Gámez-Guadix M, de Santisteban P, Resett SA (2017). Sexting among Spanish adolescents: Prevalence and personality profiles. Psichothema.

[CR25] Gordon-Messer D, Bauermeister JA, Grodzinski A, Zimmerman M (2013). Sexting among young adults. Journal of Adolescent Health.

[CR26] Hasinoff AA (2015). How to have great sext: Consent advice in online sexting tips. Communication and Critical/Cultural Studies.

[CR27] Heaven PCL, Fitzpatrick J, Craig FL, Kelly P, Sebar G (2000). Five personality factors and sex: Preliminary findings. Personality and Individual Differences.

[CR28] Hipp JR, Bauer DJ (2006). Local solutions in the estimation of growth mixture models. Psychological Methods.

[CR29] Holmes LG, Nilssen AR, Cann D, Strassberg S (2021). A sex-positive mixed methods approach to sexting experiences among college students. Computers in Human Behavior.

[CR30] Hughes DJ, Rowe M, Batey M, Lee A (2012). A tale of two sites: Twitter vs Facebook and the personality predictors of social media usage. Computers in Human Behavior.

[CR31] Jones SE, Miller JD, Lynam DR (2011). Personality, antisocial behavior, and aggression: A meta-analytic review. Journal of Criminal Justice.

[CR32] Karl K, Peluchette J, Schlaegel C (2010). Who’s posting Facebook *faux pas*? A cross-cultural examination of personality differences. International Journal of Selection and Assessment.

[CR33] Kinsey AC, Pomeroy WB, Martin CE (1948). Sexual behavior in the human female.

[CR34] Klettke B, Hallford DJ, Clancy E, Mellor DJ, Toumbourou JW (2019). Sexting and psychological distress: The role of unwanted and coerced sexts. Cyberpsychology, Behavior, and Social Networking.

[CR35] Koehn AM, Okan C, Jonason KP (2019). A primer on the dark triad traits. Australian Journal of Psychology.

[CR36] Kokkinos CM, Hatzinikolaou S (2011). Individual and contextual parameters associated with adolescents’ domain specific self-perceptions. Journal of Adolescence.

[CR37] Kokkinos CM, Antoniadou N, Markos A (2014). Cyber-bullying: An investigation of the psychological profile of university student participants. Journal of Applied Developmental Psychology.

[CR38] Kokkinos CM, Krommida C (2022). Prevalence of sexting among Greek university students: A matter of relationships?. Journal of Psychology.

[CR39] Lim MSC, Vella AM, Horyniak DR, Hellard ME (2016). Exploring attitudes towards sexting of young people: A cross-sectional study. Sexual Health.

[CR40] Liu Y, Zheng L (2020). Relationships between the Big Five, narcissistic personality traits, and online sexual activities. Personality and Individual Differences.

[CR41] Lo Y, Mendell NR, Rubin DB (2001). Testing the number of components in a normal mixture. Biometrika.

[CR42] Madigan S, Ly A, Rash CL, Van Outsyel J, Temple JR (2018). Prevalence of multiple forms of sexting behavior among youth: A systematic review and meta-analysis. JAMA Pediatrics.

[CR43] Marsh HW, Lüdtke O, Trautwein U, Morin AJS (2009). Classical latent profile analysis of academic self-concept dimensions: Synergy of person- and variable-centered approaches to theoretical models of self-concept. Structural Equation Modeling.

[CR44] Morelli M, Bianchi D, Baiocco R, Pezzuti L, Chirumbolo A (2016). Sexting, psychological distress and dating violence among adolescents and young adults. Psicothema.

[CR45] Morelli M, Chirumbolo A, Bianchi D, Baiocco R, Cattelino E, Laghi F, Sorokowski P, Misiak M, Dziekan M, Hudson HK, Marshall A, Nguyễn TT, Mark LK, Kopecký K, Szotkowski R, Demirtas ET, Ouytsel JV, Ponnet K, Drouin M (2020). The role of HEXACO personality traits in different kinds of sexting: A cross-cultural study in 10 countries. Computers in Human Behavior.

[CR46] Morin A, Litalien D (2019). Mixture modeling for lifespan developmental research.

[CR47] Mori C, Cooke JE, Temple JR, Ly A, Lu Y, Anderson N, Rash C, Madigan S (2020). The prevalence of sexting behaviors among emerging adults: A meta-analysis. Archives of Sexual Behavior.

[CR48] Muthén LK, Muthén B (2017). Mplus user's guide: Statistical analysis with latent variables, user's guide.

[CR49] Olatunde, O., & Balogun, F. (2017). Sexting: Prevalence, predictors, and associated sexual risk behaviors among postsecondary school young people in Ibadan, Nigeria. *Frontiers in Public Health,**5*. 10.3389/fpubh.2017.00096 10.3389/fpubh.2017.00096 PMC5420550 28534023

[CR50] Panayiotou G, Kokkinos MC, Spanoudis G (2004). Searching for the “Big Five” in a Greek context: The NEO-FFI under the microscope. Personality and Individual Differences.

[CR51] Roberts S, Ravn S (2020). Towards a sociological understanding of sexting as a social practice: A case study of university undergraduate men. Sociology.

[CR52] Ross MJ, Drouin M, Coupe A (2019). Sexting coercion as a component of intimate partner polyvictimization. Journal of Interpersonal Violence.

[CR53] Sesar, K., & Dodaj, A. (2019). Sexting and emotional regulation strategies among young adults. *Mediterranean Journal of Clinical Phychology,**7*. 10.6092/2282-1619/2019.7.2008

[CR54] Settles RE, Fischer S, Cyders MA, Combs JL, Gunn RL, Smith GT (2012). Negative urgency: A personality predictor of externalizing behavior characterized by neuroticism, low conscientiousness, and disagreeableness. Journal of Abnormal Psychology.

[CR55] Schreiber A, Marcus B (2020). The place of the ‘Dark Triad’ in general models of personality: Some meta-analytic clarification. Psychological Bulletin.

[CR56] Uhl CA, Rhyner KJ, Terrance CA, Lugo NR (2018). An examination of nonconsensual pornography websites. Feminism & Psychology.

[CR62] Van Ouytsel J, Van Gool E, Walrave M, Ponnet K, Peeters E (2017). Sexting: Adolescents’ perceptions of the applications used for, motives for, and consequences of sexting. Journal of Youth Studies.

[CR57] Walker K, Sleath E (2017). A systematic review of the current knowledge regarding revenge pornography and non-consensual sharing of sexually explicit media. Aggression and Violent Behavior.

[CR58] Walrave M, Heirman W, Hallam L (2014). Under pressure to sext? Applying the theory of planned behaviour to adolescent sexting. Behaviour & Information Technology.

[CR59] We Are Social. (2020). *DIGITAL 2020: GREECE*. Recovered December 9, 2020, from https://wearesocial.com/blog/2020/01/digital-2020-3-8-billion-people-use-social-media.

[CR60] Wilkinson Y, Whitfield C, Hannigan S, Azam Ali P, Hayter M (2016). A qualitative meta-synthesis of young peoples’ experiences of ‘sexting. British Journal of School Nursing.

[CR61] Wolak J, Finkelhor D (2011). Sexting: A typology.

